# Unusual massive venous hemorrhage after pancreatoduodenectomy treated by endovascular approach

**DOI:** 10.1093/jscr/rjae256

**Published:** 2024-05-14

**Authors:** Jaber Alfaifi, Cyrille Buisset, Agathe Postillon, Xavier Orry, Hervé Chanty, Adeline Germain, Ahmet Ayav

**Affiliations:** Department of Digestive, Endocrine and Metabolic Surgery, Hôpital Robert Schuman – UNEOS Groupe Hospitalier Associatif, rue du Champ Montoy, Metz 57070, France; Department of Digestive, Endocrine and Metabolic Surgery, Hôpital Robert Schuman – UNEOS Groupe Hospitalier Associatif, rue du Champ Montoy, Metz 57070, France; Department of Digestive Surgery, Hôpital Bel-Air – CHR Metz-Thionville, rue du Friscaty, Thionville 57100, France; Department of Radiology, University Hospital of Nancy, rue du Moran, Vandoeuvre-les, 54500 Nancy, France; Department of Hepatobiliary, Colorectal and Oncologic Surgery, University Hospital of Nancy, rue du Moran, Vandoeuvre-les-Nancy 54500 Nancy, France; Department of Hepatobiliary, Colorectal and Oncologic Surgery, University Hospital of Nancy, rue du Moran, Vandoeuvre-les-Nancy 54500 Nancy, France; Department of Hepatobiliary, Colorectal and Oncologic Surgery, University Hospital of Nancy, rue du Moran, Vandoeuvre-les-Nancy 54500 Nancy, France

**Keywords:** pancreaticoduodenectomy, hemorrhage, venous, surgery, endovascular, stent

## Abstract

Most post-pancreaticoduodenectomy hemorrhages (PPH) are of arterial origin, and some studies have suggested that an interventional radiology approach is most effective in reducing mortality. Venous PPH is rare, and identifying its source can be challenging. We report a case of late venous PPH in the context of a pancreatic fistula following pancreaticoduodenectomy. During surgical exploration, the area of ​​potential bleeding was inaccessible due to major inflammatory adhesions aggravated by the presence of pancreatic fistula and the delay of relaparotomy. No intra-abdominal bleeding was detected on imaging studies or during abdominal exploration; only a massive bleeding through the drain orifice, which required packing, was observed. Percutaneous transhepatic portography was performed to localize and treat the origin of the bleeding. The hemorrhage was successfully treated by endovascular approach. We found no reports in the literature on the use of interventional radiology with venous stenting to treat venous PPH, except in cases of gastrointestinal variceal hemorrhage due to portal occlusion.

## Introduction

Post-pancreatectomy hemorrhage (PPH) has a relatively low incidence but a high mortality rate [1]. Early PPH is usually the result of technical failure [2]. Late hemorrhage, with a typical delay of several days, is usually the result of surgical complications, such as ulceration at the anastomosis site, erosion of adjacent blood vessels due to a pancreatic fistula, or the development of a pseudoaneurysm [1, 2]. Currently, the first-line approach for the treatment of late arterial PPH is endovascular management [3]. Late venous hemorrhage after pancreatoduodenectomy is rare and may be difficult to treat surgically, as it is difficult to identify the source of bleeding. We report a case of a patient who presented with a major late venous PPH, which was successfully treated by an endovascular approach.

## Case report

**Figure 1:**
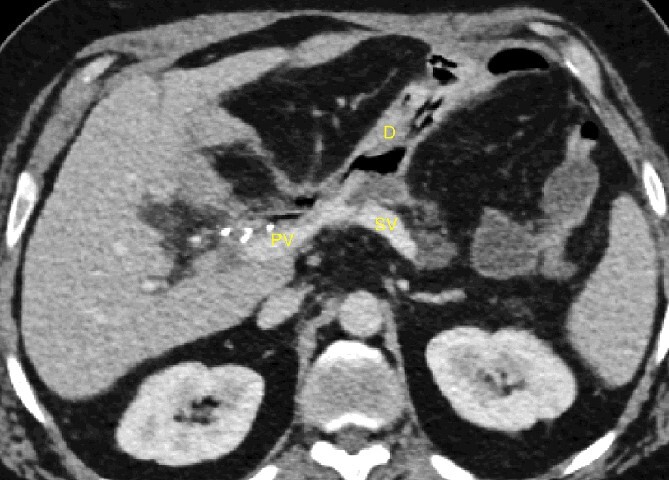
20-day post-operative CT scan showing irregularity of the splenic vein, without active bleeding. PV portal vein, SV splenic vein, D multi-tubular drain.

A 52-year-old man with a past medical history of obesity, diabetes, and Lynch syndrome, which had led to a subtotal colectomy 4 years earlier, underwent an open pancreatoduodenectomy for a duodenal adenocarcinoma in the tertiary referral center. The pancreas had a soft texture; hence, a pancreaticogastrostomy anastomosis was performed. No vascular resection or reconstruction was necessary. Multi-tubular drains were placed behind the anastomoses (pancreaticogastrostomy and hepaticojejunostomy) at the end of the procedure. Six days after surgery, the patient developed sepsis due to a pancreatic fistula (grade B, according to ISGPS definition) [4]. The patient was treated with intravenous antibiotics. On the fifteenth postoperative day, we noticed bleeding through the drain orifice (about 400 cc) that stopped spontaneously. The patient was hemodynamically stable, and no active bleeding or arterial pseudoaneurysms were found on a computed tomography (CT) scan. On the twentieth post-operative day, the hemorrhage recurred and ceased spontaneously again. The CT scan showed a splenic vein irregularity without active bleeding or intra-abdominal free fluid (Fig. 1). After a massive new bleeding the same day, we performed an exploratory laparotomy.

**Figure 2:**
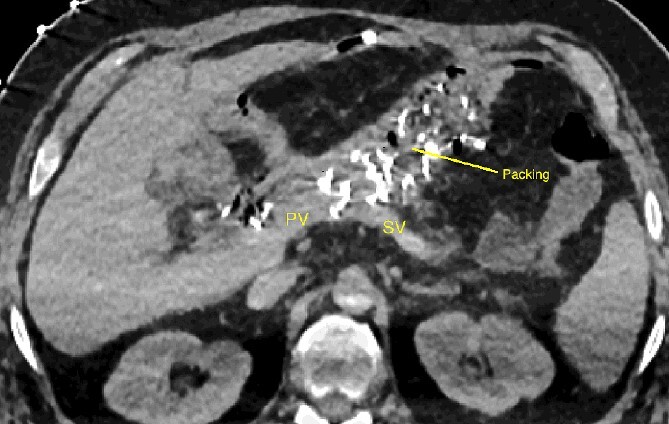
CT scan after relaparotomy located the packs used for hemorrhage control anterior to the confluence of the portal vein and splenic vein. PV portal vein, SV splenic vein.

**Figure 3:**
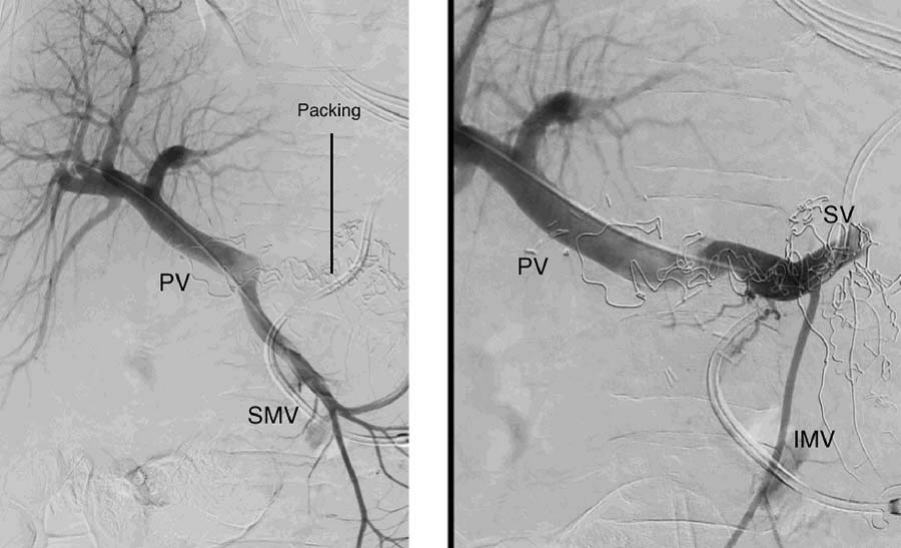
Percutaneous transhepatic portography with irregular aspect of the splenic vein behind the packs. PV portal vein, SMV superior mesenteric vein, SV splenic vein, IMV inferior mesenteric vein.

**Figure 4:**
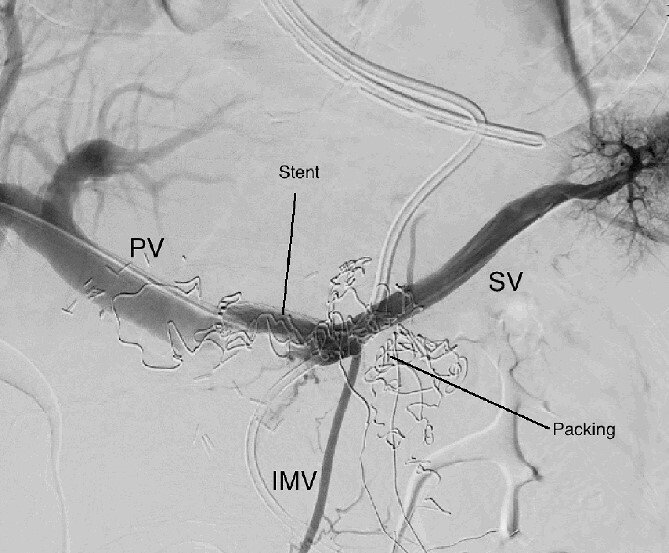
Percutaneous transhepatic portography after cover stent placement in the splenic vein behind the packs. PV portal vein, SV splenic vein, IMV inferior mesenteric vein.

**Figure 5:**
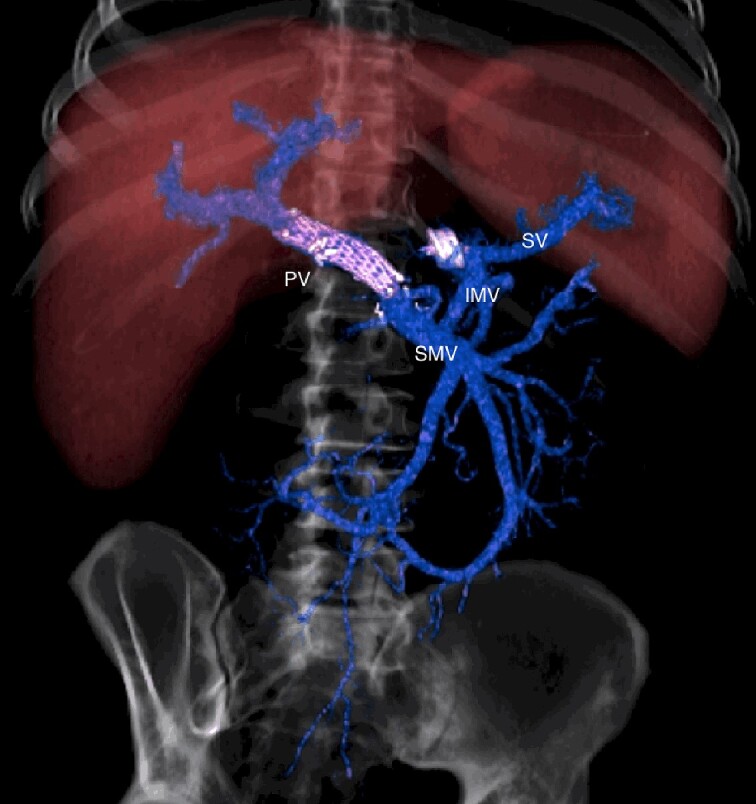
CT scan after other interventional radiology with plug in the splenic vein and cover stent in the superior mesenteric vein. PV portal vein, SMV superior mesenteric vein, SV splenic vein, IMV inferior mesenteric vein.

**Figure 6:**
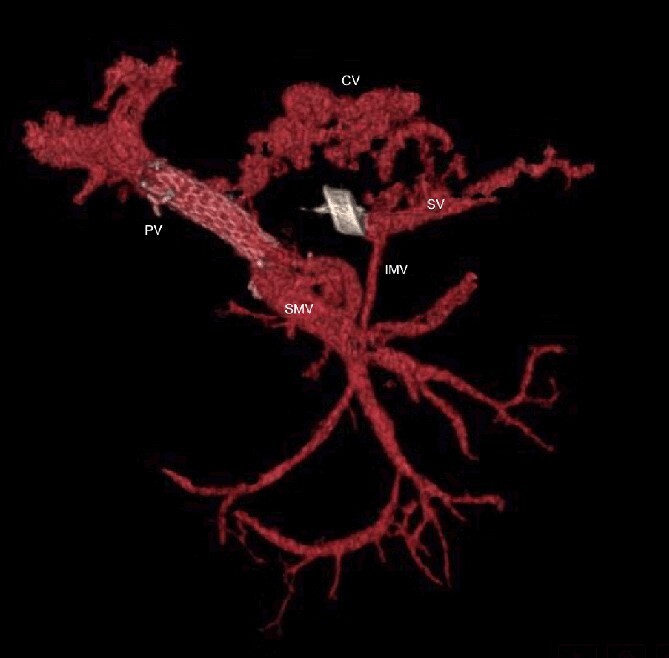
CT scan at 3 years, with development of venous collaterals that drain distal splenic vein and inferior mesenteric vein through the lesser omentum. PV portal vein, SMV superior mesenteric vein, SV splenic vein, IMV inferior mesenteric vein, CV collateral veins.

The abdominal exploration was difficult due to significant inflammatory adhesions, which can be attributed to the presence of a pancreatic fistula and the delay of relaparotomy. There was no bleeding observed within the abdominal cavity, and no bleeding was seen from the multitubular drain during the exploration. We hypothesized that the bleeding was of venous origin caused by drain-induced recurrent erosion of peripancreatic vessels, resulting in intermittent bleeding, and that removing drains could help in attaining venous hemostasis. As a result, it was decided to remove the drains. However, this resulted in a significant hemorrhage via the drain orifice. Due to failure to identify the exact origin of bleeding and without the possibility of completion pancreatectomy, we performed a damage control procedure by packing through the drain orifice. This successfully stopped the bleeding. A postoperative CT scan located the packing sponges anterior to the spleno-mesenteric confluence (SMC) (Fig. 2). A percutaneous transhepatic portography showed an irregular aspect of the SMC, without thrombi or contrast extravasation (Fig. 3). After consulting with the interventional radiology team, we placed a cover stent (Fluency™ Bard, Murray Hill, USA) in the SMC (Fig. 4). Two days later, we removed the packs, and a massive hemorrhage recurred, necessitating a new packing. The stent was not seen on a subsequent CT scan. We then performed an endovascular procedure to exclude the segment of the irregular SMC. An Amplatzer™ vascular plug (Abbott Vascular, Santa Clara, USA) was placed in the splenic vein, extending distally to just surpass the inferior mesenteric vein (IMV) insertion (Fig. 5). A cover stent was subsequently deployed in the portal vein, extending distally to the superior mesenteric vein. This allowed for the exclusion of the splenic vein segment that lies between the inferior mesenteric vein insertion point and the spleno-mesenteric confluence (Fig. 5). The packing sponges were removed 10 days later without bleeding recurrence, and the patient finally showed clinical improvement. After 3 years, the stent was visible and permeable on a follow-up CT scan. The scan showed development of venous collaterals, which drain the distal splenic vein and inferior mesenteric vein through the lesser omentum (Fig. 6).

## Discussion

Studies have shown that most delayed PPH is of arterial origin [5]. The main cause of early hemorrhage is insufficient hemostasis [6], so many authors consider relaparotomy the first line of treatment in unstable patients. In delayed PPH, the therapeutic decision is more complicated [7, 8], as pancreatic leak and sepsis in late PPH are associated with high mortality [9]. Additionally, late relaparotomy can be challenging due to the presence of inflammatory adhesions, and it is correlated with increased mortality [10].

Over the last two decades significant advances have been made in the field of endovascular interventional radiology, making it an effective alternative and decreasing mortality in PPH. Roulin *et al.* [5] found a statistically significant difference in the mortality rates of laparotomy and radiological intervention in treating delayed PPH (22% for interventional radiology vs. 47% for laparotomy; *P* = .02). Van Oosten *et al*. [11] showed that interventional angiography has a lower mortality rate than relaparotomy when used as the primary intervention for managing late postpancreatectomy hemorrhage (16% vs 37%). Many authors recommend endovascular treatment as the first reintervention option in patients with delayed PPH [12].

Venous postpancreatectomy hemorrhage is uncommon. Wellner *et al*. found that the gastrointestinal tract was the primary source of PPH, followed by the visceral artery branches and the pancreatic cut surface (30.8%, 24.4%, and 16.7%, respectively). Other extraluminal bleeding sources were found in 19% of cases, while the exact cause could not be identified in 10% [13]. Venous bleeding after pancreatoduodenectomy has been reported as gastrointestinal intraluminal bleeding. It results from variceal hemorrhage that develops secondary to portal hypertension [14]. These varices typically manifest months or even years after surgery, when postoperative scarring and inflammation cause delayed portomesenteric venous obstruction. Interventional radiology shows a high success rate in treating such cases [14].

Here we report a rare case of massive venous hemorrhage that occurred 15 days after pancreaticoduodenectomy in the context of a pancreatic fistula. We found no similar cases in the literature. Identification of bleeding of venous origin can be challenging on CT-scan. A surgical reintervention was initially attempted due to the inability to identify a bleeding source on the CT scan. The surgical exploration was extremely difficult due to major inflammatory adhesions induced by inflammation secondary to pancreatic fistula. In complex cases, identification of the bleeding with CT-scan and surgical access to the bleeding area can be very difficult and may lead to more complications. Percutaneous transhepatic portography with percutaneous transhepatic stent placement in may be an interesting option to help diagnose and treat these complex cases of delayed PPH emphasizing the role of the radiological intervention in managing PPH and should be adopted as a first option for its treatment.

## References

[1] Mañas-Gómez MJ , Rodríguez-Revuelto R, Balsells-Valls J, et al. Post-pancreaticoduodenectomy hemorrhage: incidence, diagnosis, and treatment. World J Surg 2011;35:2543–8. 21882027 10.1007/s00268-011-1222-4

[2] Wente MN , Veit JA, Bassi C, et al. Postpancreatectomy hemorrhage (PPH): an international study Group of Pancreatic Surgery (ISGPS) definition. Surgery 2007;142:20–5. 17629996 10.1016/j.surg.2007.02.001

[3] Asari S , Matsumoto I, Toyama H, et al. Recommendation of treatment strategy for postpancreatectomy hemorrhage: lessons from a single-center experience in 35 patients. Pancreatology 2016;16:454–63. 26935829 10.1016/j.pan.2016.02.003

[4] Bassi C , Marchegiani G, Dervenis C, et al. The 2016 update of the international study group (ISGPS) definition and grading of postoperative pancreatic fistula: 11 years after. Surgery 2017;161:584–91. 28040257 10.1016/j.surg.2016.11.014

[5] Roulin D , Cerantola Y, Demartines N, Schäfer M.. Systematic review of delayed postoperative hemorrhage after pancreatic resection. J Gastrointest Surg 2011;15:1055–62. 21267670 10.1007/s11605-011-1427-8

[6] Yekebas EF , Wolfram L, Cataldegirmen G, et al. Postpancreatectomy hemorrhage: diagnosis and treatment: an analysis in 1669 consecutive pancreatic resections. Ann Surg 2007;246:269–80. 17667506 10.1097/01.sla.0000262953.77735.db PMC1933568

[7] Correa-Gallego C , Brennan MF, D’Angelica MI, et al. Contemporary experience with postpancreatectomy hemorrhage: results of 1,122 patients resected between 2006 and 2011. J Am Coll Surg 2012;215:616–21. 22921325 10.1016/j.jamcollsurg.2012.07.010

[8] Feng J , Chen YL, Dong JH, et al. Post-pancreaticoduodenectomy hemorrhage: risk factors, managements and outcomes. Hepatobiliary Pancreat Dis Int 2014;13:513–22. 25308362 10.1016/s1499-3872(14)60276-9

[9] Rajarathinam G , Kannan DG, Vimalraj V, et al. Post pancreaticoduodenectomy haemorrhage: outcome prediction based on new ISGPS clinical severity grading. HPB 2008;10:363–70. 18982153 10.1080/13651820802247086 PMC2575673

[10] Standop J , Glowka T, Schmitz V, et al. Operative re-intervention following pancreatic head resection: indications and outcome. J Gastrointest Surg 2009;13:1503–9. 19421823 10.1007/s11605-009-0905-8

[11] Floortje van Oosten A , Smits FJ, van den Heuvel DAF, et al. Diagnosis and management of postpancreatectomy hemorrhage: a systematic review and meta-analysis. HPB (Oxford) 2019;21:953–61. 30962134 10.1016/j.hpb.2019.02.011

[12] Biondetti P , Fumarola EM, Ierardi AM, Carrafiello G.. Bleeding complications after pancreatic surgery: interventional radiology management. Gland Surg 2019;8:150–63. 31183325 10.21037/gs.2019.01.06 PMC6534758

[13] Wellner UF , Kulemann B, Lapshyn H, et al. Postpancreatectomy hemorrhage -- incidence, treatment, and risk factors in over 1,000 pancreatic resections. J Gastrointest Surg 2014;18:464–75. 24448997 10.1007/s11605-013-2437-5

[14] Hoffer EK , Krohmer S, Gemery J, et al. Endovascular recanalization of symptomatic portomesenteric venous obstruction after pancreaticoduodenectomy and radiation. J Vasc Interv Radiol 2009;20:1633–7. 19854066 10.1016/j.jvir.2009.09.001

